# Emotional Valence in the Eye Region Modulates the Attentional Blink in a Task-Dependent Manner: Evidence from Event-Related Potentials

**DOI:** 10.3390/brainsci12121665

**Published:** 2022-12-03

**Authors:** Shuaixia Li, Yiwen Li, Shuaicheng Liu, Weiqi He, Wenbo Luo

**Affiliations:** 1 Research Center of Brain and Cognitive Neuroscience, Liaoning Normal University, Dalian 116029, China; 2 Key Laboratory of Brain and Cognitive Neuroscience, Dalian 116029, China

**Keywords:** emotional eye region, task relevance, attentional blink, ERPs

## Abstract

Although emotional expressions conveyed by the eye regions are processed efficiently, little is known regarding the relationship between emotional processing of isolated eye regions and temporal attention. In this study, we conducted three rapid serial visual presentation (RSVP) experiments with varying task demands (emotion discrimination, eye detection, eyes ignored) related to the first target (T1) to investigate how the perception of emotional valence in the eye region (T1: happy, neutral, fearful) impacts the identification of a second target (T2: neutral houses). Event-related potential (ERP) findings indicated that fearful stimuli reliably increased N170 amplitude regardless of the emotional relevance of task demands. The P3 component exhibited enhanced responses to happy and fearful stimuli in the emotion discrimination task and to happy eye regions in the eye detection task. Analysis of T2-related ERPs within the attentional blink period revealed that T2 houses preceded by fearful and happy stimuli elicited larger N2 and P3 amplitudes than those preceded by neutral stimuli only in the emotion discrimination task. Together, these findings indicate that attention to affective content conveyed by the eyes can not only amplify the perceptual analysis of emotional eye regions but also facilitate the processing of a subsequent target.

## 1. Introduction

Due to limitations in attentional capacity, in-depth perceptual analysis requires the human brain to select visual input that is behaviorally relevant while filtering out irrelevant input, a process known as selective attention. There is considerable evidence that emotionally expressive faces have the ability to capture attention rapidly and automatically [1,2,3]. However, during the COVID-19 pandemic, requirements for face masks wearing have been implemented worldwide to aid in reducing transmission of the virus, meaning that individuals must rely on information from the eye region alone when attempting to infer another’s mental state. Indeed, increasing evidence suggests that basic facial expressions (e.g., fearful and angry) and a wide range of complex mental states (e.g., admiring and arrogant) can be recognized effectively even when the eye region is isolated [4,5,6]. In particular, fearful and angry expressions conveyed by the eye regions, which may be more significant in evolutionary terms, have been found to exert a remarkable influence on selective attention, as reflected by facilitated processing [7,8] and reliable indications of threat [9,10,11]. From the perspective of biological adaptation, this phenomenon is helpful for human survival. While it is noteworthy that happy faces conveying potential benefit are an indispensable part of our lives and can be recognized accurately from eye region cues, especially in East Asians [12], it remains unclear whether such an attentional bias can be observed for happy expressions conveyed by the eye region alone.

Several studies utilizing dot-probe tasks have demonstrated that fearful expressions in the eye region can facilitate spatial attention even when perceived unconsciously, manifesting as accelerated attentional orienting towards and delayed attentional disengagement from threat [10,11]. However, much less is known regarding the temporal dynamics of this process. To our knowledge, rapid serial visual presentation (RSVP) is the paradigm most frequently used to explore the temporal characteristics of attentional processes, in which a stream of stimuli is successively presented in the same location in rapid succession, with participants tasked with identifying two prespecified targets. A common finding in this paradigm is temporary impairment in reporting the second target (T2) when it follows the first (T1) by approximately 200–500 ms, a phenomenon referred to as the attentional blink (AB) [13,14]. Importantly, studies have indicated that the AB is reduced when emotional words [15,16], eye regions [17], full facial expressions [18,19,20], or scenes [21] are presented as T2 when emotionally neutral stimuli are presented as T1. This finding suggests that the emotional significance of T2 impacts the speed with which attention is directed to the stimulus, enabling privileged access to limited cognitive resources.

More relevant to the present situation, research investigating the effect of an emotionally valent T1 on the AB has yielded inconsistent findings. Some studies have reported that an emotional T1 enhances the AB, and that this effect is especially pronounced for affectively threatening stimuli even when they are irrelevant to the current task [22,23,24,25]. In contrast, some studies have reported no such effects, while others have even reported attenuation of AB following presentation of an emotionally valent T1 [26,27,28,29]. These discrepancies among studies may be related to the attentional demands associated with T1 processing. To address this issue, Stein, Zwickel, Ritter, Kitzmantel, and Schneider [29] used images of fearful and neutral faces as the T1 stimuli and images of two neutral scenes as the T2 stimuli. Participants were asked to attend to different features of the T1 face in three tasks (emotional judgment, gender judgment, and passive viewing task). Behavioral results indicated that fearful faces enhanced the AB effect only in the emotional judgment task, providing initial evidence for the impact of the task demands of emotional T1 on AB. Nonetheless, it remains unknown how and precisely when task demands associated with emotional T1 exert their influence on the processing of T1 and T2. To address this issue in the present study, we employed an event-related potential (ERP) technique to investigate how feature-based attention to emotional eye regions affects the processing of T2 by manipulating the task demands of T1.

Given its advantages in terms of temporal resolution, many studies have utilized ERP to investigate the temporal dynamics underlying the perception of facial expressions. There is growing consensus that the N170 and P3 are engaged in the early perceptual encoding and late elaborated processing of emotional faces [3,30,31]. Originally, the N170 was thought to reflect the structural analysis and integration of faces given relative increases in its amplitude for faces vs. objects [32]. When compared with the whole face or special facial regions such as the nose or mouth, isolated eye regions elicit increases in N170 amplitude and delays in N170 latency, reflecting the recruitment of both eye- and face-selective cells [33,34,35,36]. Furthermore, this component has been associated with modulating emotional facial expressions [30]. Emotional whole faces or isolated eye regions, especially those signaling potential threats, can automatically evoke augmented potentials when compared with neutral ones. This phenomenon has been observed across a range of tasks [1,2,37], image property [17,38,39,40,41], and manipulations of available attentional resources [19,42,43], confirming that threatening stimuli are processed with priority. Other studies have also reported an increased N170 response for facial expressions over the right hemisphere when compared with that in the left [19,37], supporting the notion of right-hemispheric superiority.

The late P3 component, which is believed to be linked to sustained elaborate analysis and the consolidation of affectively meaningful stimuli in working memory, is largely influenced by top-down attention and task demands at hand [31,44]. It has been well documented that faces with high emotional or motivational significance, such as fearful, angry, and happy faces, can produce relative increases in P3 amplitude when compared with responses for neutral faces, especially when feature-based attention is guided to the affective content of a face [19,41,45]. Similarly, several studies have reported enhanced P3 for threatening eye regions (i.e., fearful and angry) relative to neutral ones [17,46]. Notably, preliminary evidence investigating the neural correlates of emotional AB and emotion-induced blindness indicates that negative faces or scenes used as either the T1 or to-be-ignored distractors are usually associated with increased P3 activity, while the P3 amplitude evoked by a subsequent neutral target is significantly suppressed. These findings are consistent with the idea of an impairment in the representation of information at the level of working memory consolidation [47,48]. Nevertheless, Jia, Zhang, and Zhang [24] reported significant enhancement of the P3 amplitude for fearful T1 and neutral T2 stimuli during the AB period when compared with those observed in the neutral T1 condition. The authors explained that this effect may be related to negativity bias and overinvestment of attentional resources. These incongruent findings highlight the need to further explore the effects of task demands and emotional stimuli on the neural correlates of AB.

Apart from the P3, the earlier posterior N2—a visual component that is often referred to as the early posterior negativity component in studies of emotion perception—is occasionally assumed to be engaged in the conscious detection and representation of T2 stimuli in a limited-capacity system, especially when a dual-target rapid serial visual presentation (RSVP) task is used. Correct recognition of T2 leads to an augmented N2 amplitude when compared with that observed for unidentified T2s [48,49,50]. Additional studies examining emotion-induced blindness have reported a pronounced trade-off in the amplitude of the N2s associated with a task-irrelevant emotional distractor and a task-relevant neutral target. Such studies have reported that increases in N2 elicited by an emotionally negative distractor are accompanied by an attenuated N2 response immediately following presentation of the target. This finding suggests that an emotionally arousing distractor can capture and hold attention automatically, leading to suppressed attentional engagement with targets [47].

**Figure 1:**
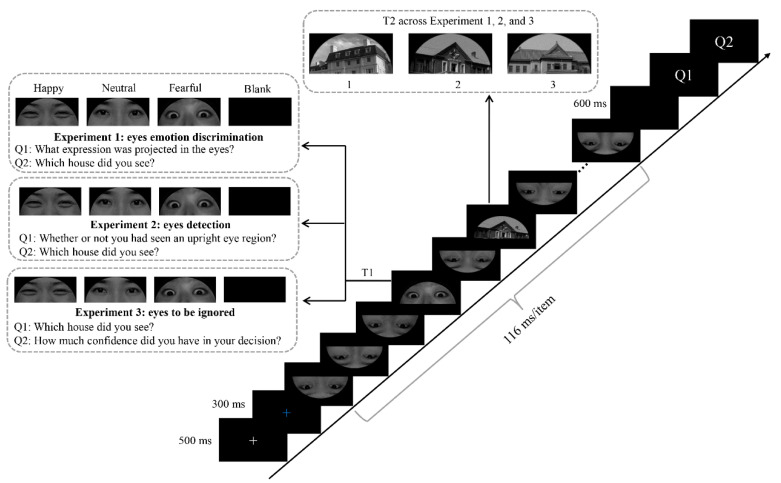
Experimental design and diagram of a representative trial.

Considering the fundamental role of human eyes in social communication, the present study aimed to explore the influence of task demands on the processing of emotionally valent eye region stimuli, as well as their modulatory influence on the AB effect. To achieve this aim, we measured ERPs while participants completed a set of three RSVP experiments, in which upright happy, neutral, or fearful eye region stimuli were presented as the T1 stimuli or to-be-ignored salient distractors. In these experiments, three upright neutral houses were used as the T2 stimuli, and images of inverted neutral eye regions were used as distractors. The number of distractors between T1 and T2 was set to either 1 or 5, resulting in T2 appearing at a lag of 2 or 6 (stimulus onset asynchrony was 232 or 696 ms). The experimental protocol and stimuli in the three experiments were kept constant other than the task demands related to T1, as shown in Figure 1 (Experiment 1: emotional categorization; Experiment 2: eye region detection; Experiment 3: to-be-ignored salient distractors). In line with findings from Stein, Zwickel, Ritter, Kitzmantel, and Schneider [29], we hypothesized that the AB effect would vary as a function of task demands related to T1. According to the ERP patterns associated with processing emotionally arousing stimuli outlined above, we further expect the early N170 to reflect preferential and task-independent processing for fearful eye regions and the late P3 to reflect task-dependent responses to emotionally valent eye region stimuli during the emotional categorization task, manifesting as significant increases in amplitude for emotional eye regions relative to neutral regions. We also hypothesized that different N2 and P3 responses to T2 houses would occur following emotional and neutral T1 only during the emotional categorization task.

## 2. Materials and Methods

### 2.1. Participants

In each of three experiments, 24 undergraduate students from the Liaoning Normal University took part (experiment 1: 4 males, mean age = 20.92 years; experiment 2: 8 males, mean age = 20.58 years; experiment 3: 11 males, mean age = 21.12 years) and received cash payment for their participation. This sample size can be used to investigate the effect of emotional eye region on AB at the medium effect size level (Cohen *f* = 0.25, power = 0.90, α error = 0.05) on the basis of a priori power analysis in G*Power 3.1 (Franz Faul, Uiversität Kiel, Germany) [51]. All participants were right-handed, had normal or corrected-to-normal vision, and reported the absence of any neurological or psychiatric disorder. Informed consent in line with the university’s ethical guidelines was obtained before starting the experiment.

### 2.2. Stimuli

Fourteen items (12 distractors and 2 targets) were presented in each RSVP stream. The T1 stimuli included six eye region images cropped from 2 fearful, 2 neutral, and 2 happy face models included in the Chinese Facial Affective Picture System (CFAPS) [52]. Another 12 neutral eye region images were selected from the same face set and presented inversely as distractor stimuli. The T2 stimuli included three house images selected from the Chinese Affective Picture System (CAPS) [53]. All stimuli were cropped to a semi-elliptical shape using Adobe Photoshop 8.0 software, and the size was adjusted to 252 pixels in width and 100 pixels in height, subtending a visual angle of 5.9° × 2.3° at a 65 cm viewing distance. In view of the potential influence of perceptual similarity between targets and distractors on AB [54], studies investigating the effect of facial expressions on AB have often used inverted neutral faces or eye regions as distractors [17,19,23]. Accordingly, inverted images of neutral eye regions were used as distractors during the present study. However, the inverted distractors were presented in a shape different from the targets to reduce perceptual difficulty and avoid the occurrence of a floor effect. In our prior study [17], we evaluated valence and arousal for these eye regions systematically. Significant differences in valence were observed among the three valences (M ± SD, happy: 8.08 ± 0.80, neutral: 4.10 ± 0.99, fearful: 1.81 ± 1.05; *F*(2, 46) = 284.55, *p* < 0.001, *η_p_*^2^ = 0.93), and there was also a significant main effect of arousal rating [*F*(2, 46) = 91.73, *p* < 0.001, *η_p_*^2^ = 0.80]. Happy (7.27 ± 1.33) and fearful (8.12 ± 1.62) eye regions were rated as more arousing than neutral ones (3.27 ± 1.25, *ps* < 0.001), with no differences between the former two conditions (*p* > 0.09).

### 2.3. Procedure

The experiment was conducted in a room with attenuated sound and soft lighting. Participants were comfortably seated in a chair positioned 65 cm from the screen. The stimuli were displayed on a 19-inch computer monitor with a refresh rate of 60 Hz. E-Prime 2.0 software (Psychology Software Tools, Inc., Pittsburgh, PA, USA) was used to present stimuli and collect response data.

In each trial, a white central fixation cross was presented for 500 ms, following which a blue central fixation cross was presented for 300 ms. An RSVP stream consisting of 14 items (12 distractors, T1, and T2) was then presented in the center of the screen (Figure 1). Each item in the stream was displayed for 116 ms and was immediately followed by the subsequent item. There were two possible lags (lag2 and lag6) between T1 and T2; the lag2 condition referred to one intervening distractor item between two targets, while the lag6 condition referred to five intervening distractor items between the two targets. T1 was displayed in serial position 3, 4, or 5 of the 14-item stream in a randomized and equally probabilistic manner. To exclude the interference of ERPs induced by distractors presented successively and obtain pure ERPs related to T1, we introduced a control condition in which the T1 was replaced with a black screen while other items in the stream remained constant, as in previous studies [19,24,55]. A 600 ms blank interval was presented after the stream offset.

Then, participants were asked to report the specific classification of the two targets by responding to two questions. For Question 1 in Experiment 1, participants were asked whether they had seen an upright eye region, and if so, to pinpoint what emotion was depicted in the eye region. Responses were entered by typing “1” for “happy”, “2” for “neutral”, “3” for “fearful”, and “0” for “eye region absent”. In Question 2, participants were asked to indicate which house they had seen. Responses were entered by typing “1” for “house 1”, “2” for “house 2”, and “3” for “house 3”. For Question 1 in Experiment 2, participants were asked whether they had seen an upright eye region. Responses were entered by typing “1” for yes and “2” for “no”. Question 2 was the same as that in Experiment 1. For Question 1 in Experiment 3, participants were asked to report which house they had seen as T2. Responses were entered by typing “1” for “house 1”, “2” for “house 2”, and “3” for “house 3”. For Question 2, participants were asked to report their level of confidence in the previous judgement. Responses were entered by typing “1” for “very sure”, “2” for sure”, and “3” for “unsure”. Following a 600 ms blank interval, the next trial appeared. Twenty-four trials were presented in the practice phase to ensure that participants clearly understood the experimental procedure. Each session encompassing the three experiments included 576 trials, which were presented in 6 blocks of 96 trials each. In each block, different conditions with equal numbers of trials were presented in a counterbalanced order.

### 2.4. Recording and Analysis of EEG Data

Electrophysiological activity was recorded from 64 scalp sites using tin electrodes mounted on an elastic cap in accordance with the extended 10–20 system (Brain Products, Munich, Germany), with the reference electrode located at FCz. An electrode situated 10 mm below the right eye was also used to record vertical electrooculograms (VEOG). Impedances of all electrodes were kept below 5 kΩ. Continuous EEG signals were filtered with a band-pass of 0.01–100 Hz and sampled at a rate of 500 Hz/channel.

Continuous EEG signals were re-referenced to an average reference. Major artifacts such as eye blinks and ocular movements were corrected using the ocular correction ICA procedure [56] implemented in Brain Vision Analyzer 2.0 software (Brain Products, Gilching, Germany). After applying a low-pass filter at 30 Hz, the data were segmented into epochs from 200 ms before to 1000 ms after T1 stimulus onset. Baseline correction was performed using a 200 ms interval prior to T1 onset, following which trials containing eye blinks, eye movements, or other artifacts exceeding ± 80 μV were excluded. The remaining trials with correct responses to both questions were averaged for each participant in each condition. Overall, the number of valid trials used for averaging were 66.21 ± 5.19 (M ± SD), 60.62 ± 8.18, 63.87 ± 6.10, 63.12 ± 8.78, 69.29 ± 3.25, 67.29 ± 3.25, 68.37 ± 3.36, and 67.21 ± 5.34 for the happy-lag2, neutral-lag2, fearful-lag2, control-lag2, happy-lag6, neutral-lag6, fearful-lag6, and control-lag6 conditions in Experiment 1, respectively. Correspondingly, there were 69.87 ± 2.94 (happy-lag2), 69.13 ± 4.54 (neutral-lag2), 68.96 ± 3.96 (fearful-lag2), 64.63 ± 4.35 (control-lag2), 70.50 ± 1.74 (happy-lag6), 70.25 ± 2.34 (neutral-lag6), 70.29 ± 2.53 (fearful-lag6), and 68.75 ± 3.67 (control-lag6) trials averaged for each condition in Experiment 2. Lastly, there were 69.00 ± 4.18 (happy-lag2), 68.92 ± 3.92 (neutral-lag2), 68.21 ± 5.00 (fearful-lag2), 69.08 ± 4.31 (control-lag2), 69.42 ± 4.15 (happy-lag6), 69.04 ± 4.25 (neutral-lag6), 68.92 ± 4.32 (fearful-lag6), and 69.21 ± 4.27 (control-lag6) trials averaged for each condition in Experiment 3. Additionally, a subtraction technique [55] was used to subtract the average waveform of the control condition from the happy, neutral, or fearful conditions separately to calculate pure brain activities elicited by different types of T1 stimuli for each participant.

In previous studies [24,47], the mean amplitudes of the N170 and P3 components were measured separately. To explore the emotional effect of T1 on T2 house perception, we additionally analyzed the mean amplitudes of N2 and P3 components evoked by T2 under the lag2 condition. A collapsed localizer approach was used to first average the waveforms across all participants and all conditions, allowing for precise detection and extraction of the temporal and topographical distributions of a given component from the generated collapsed waveforms. This method was applied to define the time ranges and electrode sites for specific components as previously described [57]. In this regard, the eye region related to the N170 component was measured from the electrode sites of PO7, PO8, P7, and P8 within the range of 228–258 ms. The P3 component was analyzed at a set of centro-parietal electrodes (FC3, FCz, FC4, C3, Cz, C4, CP3, CPz, CP4) within the range of 400–500 ms. To assess components related to T2 houses in the lag2 condition, the N2 was measured at PO7, PO8, P7, and P8 electrodes within 435–475 ms following the presentation of the T1 stimulus. The following P3 still held the maximum amplitudes at electrode sites containing FC3, FCz, FC4, C3, Cz, C4, CP3, CPz, and CP4 within the time window of 532–632 ms post-T1 stimulus. Although the time windows of these components lagged behind those typically reported, we believe they were normal for three reasons. Firstly, the scalp topographies at these time ranges were consistent with expectations for traditional ERPs. Secondly, research has indicated that an isolated eye region can elicit a larger and more delayed N170 than a normal face given the involvement of higher-order cognitive processes, such as the eye region detector and neural inhibition mechanisms [34,35]. Thirdly, the dual-target RSVP task is characterized by an attentional challenge, which is associated with delays in the ERP time window [17,24,47].

The above components were analyzed using the same strategy in all three experiments. Two-way repeated measures analysis of variance (ANOVA) was used to evaluate T2 accuracy data in each experiment, using the eye region valence (3 levels: happy, neutral, fearful) and lag (2 levels: lag2, lag6) as within-subject factors. Two-way repeated measures ANOVA was also used to examine the mean amplitudes of the N170, N2, and P3 components using eye region valence (3 levels: happy, neutral, fearful) and hemisphere (2 levels for N170 and N2: left, right; 3 levels for P3: left, middle, right) as within-subject factors. The control condition (eye region absent) designed to purify the ERPs implicated in T1 processing was excluded from the data analysis given its lack of research value [28]. Degrees of freedom and *p* values were adjusted according to the Greenhouse–Geisser epsilon correction, and Bonferroni correction (*p* < 0.05) was used for post hoc comparisons. The partial eta squared (*η_p_*^2^) was reported as a measure of the effect size.

## 3. Results

### 3.1. Experiment 1

#### 3.1.1. Behavioral Results

A one-way repeated measures ANOVA on T1 accuracy revealed a significant main effect of eye region valence [*F*(2, 46) = 6.86, *p* = 0.002, *η_p_*^2^ = 0.23]. Post hoc comparisons indicated that accuracy was higher for happy trials (M ± SE, 0.98 ± 0.01) than for neutral trials (0.96 ± 0.01, *p* = 0.006), while differences between other conditions were not significant (fearful: 0.97 ± 0.01, *ps* ≥ 0.08).

T2 accuracy was only measured for trials in which T1 was correctly identified, since the source of T2 error in T1-incorrect trials is unclear [54]. A two-way repeated measures ANOVA on T2 accuracy yielded significant main effects of lag and T1 valence [*F*(1, 23) = 33.81, *p* < 0.001, *η_p_*^2^ = 0.59; *F*(2, 46) = 23.21, *p* < 0.001, *η_p_*^2^ = 0.50]. Accuracy was higher for lag6 (M ± SE, 0.96 ± 0.01) than lag2 (0.90 ± 0.01, *p* < 0.001). T2 houses presented in the happy (0.96 ± 0.01, *p* < 0.001) and fearful (0.93 ± 0.01, *p* = 0.004) T1 conditions were reported more accurately than those in the neutral T1 condition (0.91 ± 0.01), and the difference between the former two conditions was significant (*p* = 0.002). Furthermore, there was a significant interaction between lag and T1 valence [*F*(2, 46) = 7.61, *p* = 0.001, *η_p_*^2^ = 0.25]. Post hoc tests indicated that T2 accuracy decreased gradually from the happy T1 (0.94 ± 0.01) to fearful T1 (0.90 ± 0.02, *ps* = 0.004) and then to the neutral T1 (0.86 ± 0.02, *ps* ≤ 0.004) trials under the lag2 condition. T2 accuracy significantly differed between happy T1 trials (0.97 ± 0.01) and neutral T1 trials (0.95 ± 0.01, *ps* = 0.008) under the lag6 condition, although there were no differences between any other conditions (fearful: 0.96 ± 0.01, *ps* > 0.17).

#### 3.1.2. ERP Results

##### ERPs Related to the T1 Eye Region

**Figure 2:**
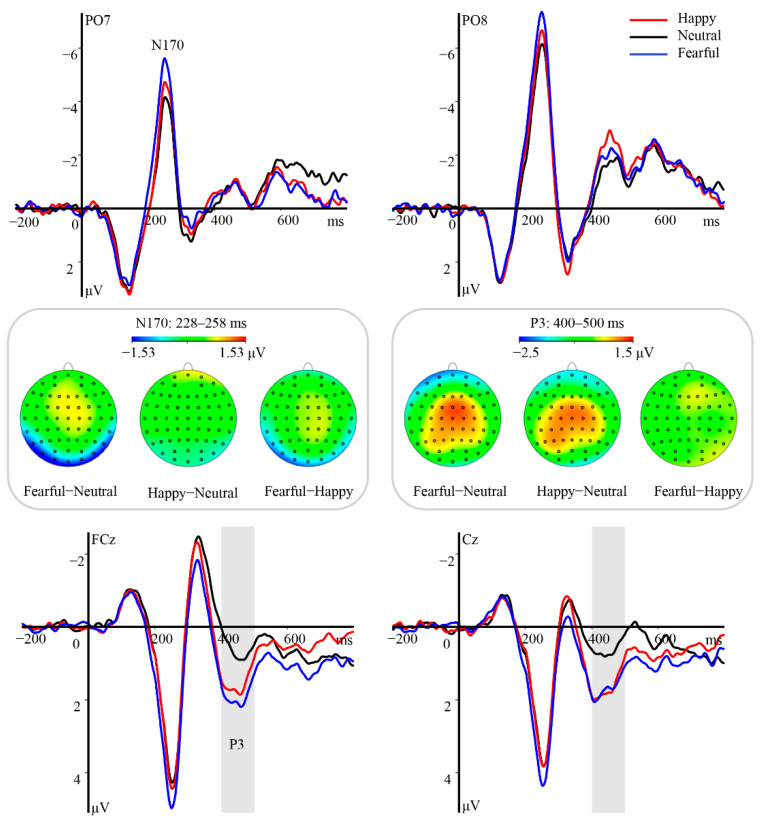
Modulation of the N170 and P3 components in response to emotional valence of eye region stimuli in Experiment 1. Top and bottom panels: Grand average ERP waveforms of N170 and P3 for happy (red line), neutral (black line), and fearful (blue line) eye regions at the indicated electrodes. The gray area indicates the time window of the P3 component (400–500 ms). Middle panel: Topographical difference maps depicting the effect of emotional valence in the eye region on the time windows for N170 and P3.

Statistical analyses of N170 revealed a significant main effect of hemisphere [*F*(1, 23) = 31.09, *p* < 0.001, *η_p_*^2^ = 0.57]. Post hoc tests indicated that the amplitude was more negative over the right hemisphere (M ± SE, −6.72 ± 0.72 μV) than over the left hemisphere (−3.95 ± 0.54, *p* < 0.001). Moreover, this component varied as a function of emotional valence [*F*(2, 46) = 19.48, *p* < 0.001, *η_p_*^2^ = 0.46], with amplitudes gradually decreasing from fearful (−6.10 ± 0.60 μV, *ps* ≤ 0.003) to happy (−5.28 ± 0.58 μV, *ps* ≤ 0.041) and then to neutral eye regions (−4.62 ± 0.63 μV, Figure 2). However, the interaction between emotional valence and hemisphere was not significant [*F*(2, 46) = 0.49, *p* = 0.62, *η_p_*^2^ = 0.02].

The analysis of the P3 yielded a pronounced main effect of emotional valence [*F*(2, 46) = 25.98, *p* < 0.001, *η_p_*^2^ = 0.53]. Both the happy (M ± SE, 1.30 ± 0.28 μV) and fearful eye region (1.31 ± 0.28 μV) stimuli elicited significantly larger amplitudes than neutral stimuli (0.43 ± 0.27 μV, *ps* < 0.001), while the difference in amplitude between the former two conditions did not reach statistical significance (*p* = 1, Figure 2). Furthermore, the interaction between emotional valence and hemisphere was significant [*F*(4, 92) = 5.50, *p* = 0.002, *η_p_*^2^ = 0.19]. Post hoc tests revealed prominent amplitude differences between emotional and neutral eye regions at all regions of interest (*ps* ≤ 0.004). No main effect of hemisphere was observed [*F*(2, 46) = 0.35, *p* = 0.71, *η_p_*^2^ = 0.01].

##### ERPs Related to T2 Houses

**Figure 3:**
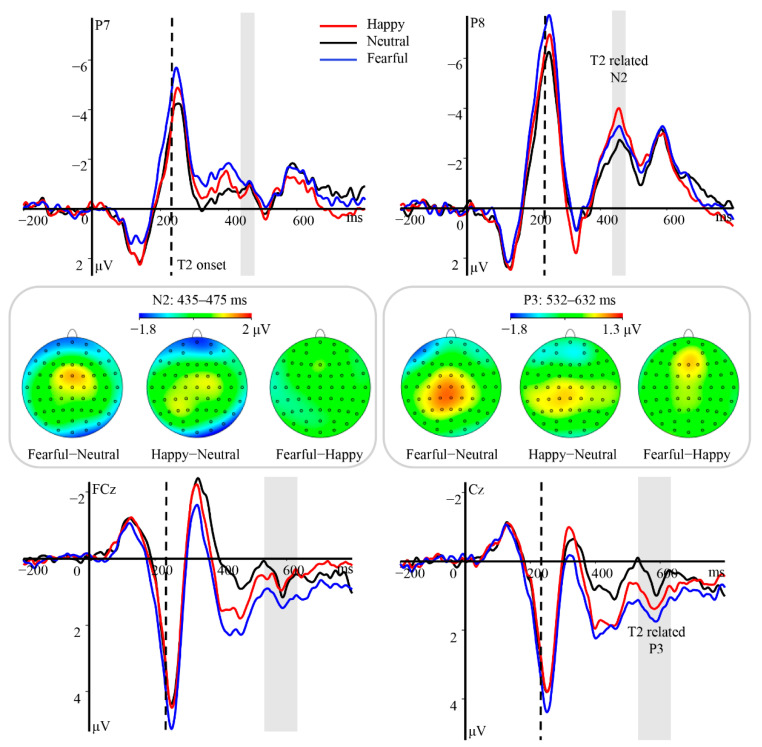
Main effects of T1 valence on T2-related N2 and P3 components. Top and bottom panels: Grand average ERP waveforms for N2 and P3 for T2 houses in the happy (red line), neutral (black line), and fearful (blue line) conditions at the indicated electrodes. Gray areas indicate the time windows of N2 and P3. Middle panel: Topographical difference maps depicting the emotional valence effect for T2 houses within the AB period during the time windows for N2 and P3.

The analysis of the N2 component revealed a significant main effect of hemisphere [*F*(1, 23) = 8.51, *p* = 0.008, *η_p_*^2^ = 0.27], with enhanced amplitudes over the right hemisphere (M ± SE, −2.13 ± 0.62 μV) relative to the left hemisphere (−0.67 ± 0.57 μV, *p* = 0.008). Furthermore, there was a significant main effect of T1 valence [*F*(2, 46) = 3.76, *p* = 0.031, *η_p_*^2^ = 0.14]; however, pair-wise comparisons did not reveal any amplitude differences for this component (*ps* > 0.12). The interaction between hemisphere and T1 valence also reached statistical significance [*F*(2, 46) = 4.97, *p* = 0.019, *η_p_*^2^ = 0.18]. Houses in the happy (−2.66 ± 0.74 μV, *p* = 0.009) and fearful (−2.30 ± 0.69 μV, *p* = 0.04) T1 trials elicited larger N2 amplitudes over the right hemisphere than did those in neutral T1 trials (−1.43 ± 0.50 μV; Figure 3), with no differences between the former two conditions (*p* = 0.67). No differences in N2 amplitude over the left hemisphere were observed among the conditions (happy: −0.70 ± 0.70 μV, neutral: −0.45 ± 0.46 μV, fearful: −0.86 ± 0.62 μV, *ps* > 0.50).

The P3 component was modulated by T1 valence [*F*(2, 46) = 10.59, *p* < 0.001, *η_p_*^2^ = 0.32], with more pronounced positivity for houses in the fearful (0.98 ± 0.34 μV, *p* = 0.002) and happy (0.80 ± 0.33 μV, *p* = 0.018) trials than in the neutral trials (0.30 ± 0.31 μV; Figure 3). There was also a significant interaction between T1 valence and hemisphere [*F*(4, 92) = 2.82, *p* = 0.029, *η_p_*^2^ = 0.11]. Further analyses revealed increases in P3 amplitude over the right hemisphere in response to houses in the happy (0.93 ± 0.54 μV) and fearful T1 (1.40 ± 0.55 μV) trials when compared with responses observed in neutral trials (0.19 ± 0.47 μV, *p* ≤ 0.05). However, no such differences were observed over the middle region or left hemisphere (*ps* > 0.09), and no main effect of hemisphere was observed [*F*(2, 46) = 0.51, *p* = 0.60, *η_p_*^2^ = 0.02].

In summary, the results of Experiment 1 (explicit emotional task) indicated that images of both happy and fearful eye regions presented as T1 stimuli are processed efficiently and facilitate the discrimination of a subsequent target within the AB period, resulting in an attenuated AB. This effect replicates the results of previous studies examining the emotional impact of T2 on AB [17,18,19]. At the neural level, both the N170 and P3 components varied as a function of the valence conveyed by the eye region, with enhanced amplitudes for fearful and happy eye regions relative to neutral ones. Moreover, fearful eye regions elicited a larger N170 response than happy eye regions. Previous studies have demonstrated that emotional facial expressions with higher evolutionary significance can reliably elicit larger N170 amplitudes than neutral expressions, reflecting the priority with which affective faces are processed [30,31,41]. Moreover, the P3 has been implicated in sustained attention and elaborative evaluation of emotionally relevant stimuli [44,58]. Both positive and negative faces have been found to capture more attentional resources and produce increases in P3 amplitudes when compared with neutral faces [19,59]. In line with these findings, the present results support the notion that stimuli depicting fearful and happy eye regions can attract attention preferentially and facilitate elaborative evaluation at distinct processing stages.

In the analysis of ERPs associated with T2 in the lag2 condition, we observed increased amplitudes of the posterior N2 and P3 components over the right hemisphere for houses preceded by fearful and happy eye region stimuli, when compared with amplitudes observed for those preceded by neutral stimuli. This response pattern is partially congruent with prior research conducted by Jia, Zhang, and Zhang [24], who used fearful and neutral faces as T1 and found significantly enhanced amplitudes of P3s associated with fearful T1 and subsequent T2 when compared with those observed in the neutral condition. More importantly, in our recent studies that employed emotional eye region stimuli as T2, we observed enhanced P3 amplitudes for fearful and happy eye regions when compared with those observed for neutral stimuli, indicating that emotional eye regions are effectively represented in working memory even under limited attentional resources [17]. Therefore, enhancement of the N2 and P3 amplitudes by T2 in the fearful and happy T1 conditions seem to reflect an overinvestment of attentional resources caused by elaborated T1 processing, in turn resulting in increased representation in working memory.

### 3.2. Experiment 2

#### 3.2.1. Behavioral Results

In this expression-irrelevant task, we observed no main effect of eye region valence on T1 accuracy [*F*(2, 46) = 1.01, *p* = 0.37, *η_p_*^2^ = 0.04]. However, we observed a significant main effect of lag on T2 accuracy [*F*(1, 23) = 4.42, *p* = 0.047, *η_p_*^2^ = 0.16], which was higher in the lag6 condition (M ± SE, 0.99 ± 0.01) than in the lag2 condition (0.98 ± 0.01, *p* = 0.047). However, there was no significant main effect of T1 valence [*F*(2, 46) = 2.59, *p* = 0.09, *η_p_*^2^ = 0.10] or interaction between lag and T1 valence [*F*(2, 46) = 0.47, *p* = 0.63, *η_p_*^2^ = 0.02].

#### 3.2.2. ERP Results

##### ERPs Related to the T1 Eye Region

**Figure 4:**
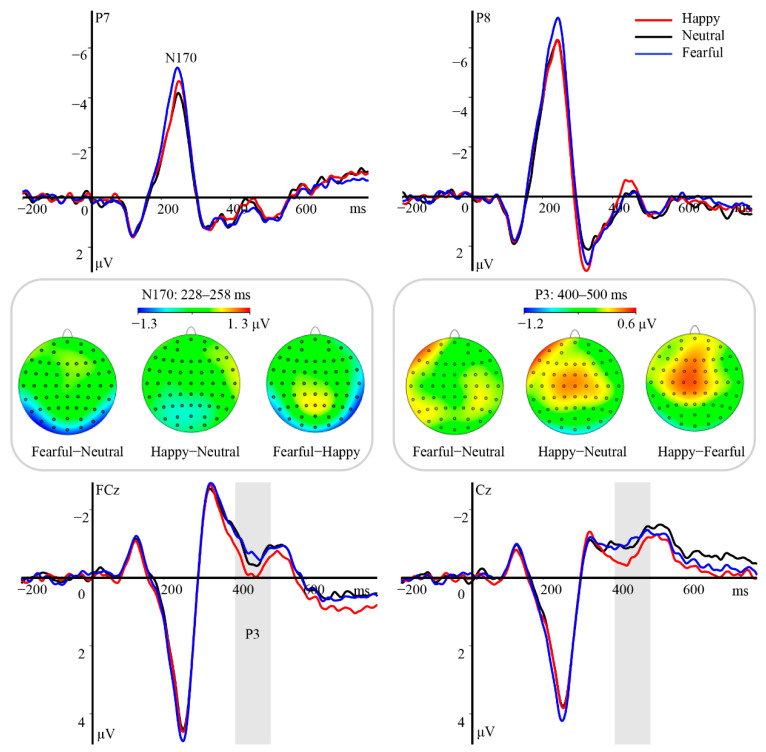
Main effects of emotional valence of eye regions on N170 and P3 amplitudes in Experiment 2. Top and bottom panels: Grand average ERP waveforms of N170 and P3 for happy (red line), neutral (black line), and fearful (blue line) eye regions at the indicated electrodes. Gray area indicates the time window for the P3 component (400–500 ms). Middle panel: Topographical difference maps depicting the effect of emotional valence in eye regions during the time windows for N170 and P3.

Our findings indicated that the emotional valence of the eye region modulated the amplitude of the N170 component [*F*(2, 46) = 10.68, *p* < 0.001, *η_p_*^2^ = 0.32]. N170 amplitudes were higher for fearful eye regions (M ± SE, −6.15 ± 0.54 μV) than for happy (−5.39 ± 0.56 μV, *p* = 0.008) or neutral (−5.15 ± 0.60 μV, *p* = 0.002) ones, while the difference in amplitude between the latter two conditions was not significant (*p* = 0.63; Figure 4). Furthermore, there was a significant main effect of hemisphere [*F*(1, 23) = 27.95, *p* < 0.001, *η_p_*^2^ = 0.55]. When compared with that observed over the left hemisphere (−4.51 ± 0.49 μV), the N170 amplitude was enhanced over the right hemisphere (−6.62 ± 0.67 μV, *p* < 0.001). However, the interaction between hemisphere and emotional valence was not significant [*F*(2, 46) = 0.44, *p* = 0.64, *η_p_*^2^ = 0.02].

Our analysis also revealed significant main effects of hemisphere [*F*(2, 46) = 6.08, *p* = 0.005, *η_p_*^2^ = 0.21] and emotional valence [*F*(2, 46) = 6.40, *p* = 0.004, *η_p_*^2^ = 0.22] on the P3 component. Follow-up tests indicated that amplitudes were higher for happy eye regions (M ± SE, −0.18 ± 0.25 μV) than for neutral (−0.44 ± 0.24 μV, *p* = 0.032) or fearful (−0.47 ± 0.26 μV, *p* = 0.01) eye regions, with no differences between the latter two conditions (*p* = 1; Figure 4). Moreover, the P3 amplitudes were more pronounced over the left (−0.14 ± 0.25 μV, *p* = 0.016) and right (0.02 ± 0.24 μV, *p* = 0.011) hemispheres than over the middle region (−0.92 ± 0.36 μV). Nevertheless, the interaction between emotional valence and hemisphere was not significant [*F*(4, 92) = 1.43, *p* = 0.23, *η_p_*^2^ = 0.06].

##### ERPs Related to T2 Houses

In the analysis of the N2 component, we observed a significant main effect of hemisphere [*F*(1, 23) = 8.64, *p* = 0.007, *η_p_*^2^ = 0.27], with more pronounced negativity over the right hemisphere (−1.08 ± 0.63 μV) than over the left hemisphere (−0.01 ± 0.57 μV, *p* = 0.007). However, neither the main effect of T1 valence [*F*(2, 46) = 1.09, *p* = 0.34, *η_p_*^2^ = 0.04] nor the interaction between hemisphere and T1 valence [*F*(2, 46) = 1.21, *p* = 0.31, *η_p_*^2^ = 0.05] reached statistical significance.

For the P3 component, there were no significant main effects of hemisphere [*F*(2, 46) = 0.01, *p* = 0.99, *η_p_*^2^ < 0.001], T1 valence [*F*(2, 46) = 1.67, *p* = 0.20, *η_p_*^2^ = 0.07], or interaction between them [*F*(4, 92) = 0.64, *p* = 0.63, *η_p_*^2^ = 0.03].

To summarize, participants in Experiment 2 were asked to focus on the emotionally irrelevant information (i.e., the presence of an upright eye region) of T1 as well as the category of T2. At the behavioral level, only the main effect of lag was significant, with higher accuracy for lag6 than for lag2 condition. These results align with those for Experiment 1 and with the well-accepted notion that stimulus processing under adequate attentional resources is highly efficient and associated with excellent task performance [54]. It must be noted that the significant interaction between T1 valence and lag in Experiment 1 was absent in Experiment 2. Previously, Stein, Zwickel, Ritter, Kitzmantel, and Schneider [29] manipulated participants’ attention to the gender of T1 faces and reported an absence of AB modulation by fearful faces. Consistent with their findings, the present data suggest that intentional decreases in attention to the emotionally relevant features of the facial stimuli can eliminate the prominent AB.

At the electrophysiological level, our findings indicated that the N170 and P3 components were modulated by the emotional valence of the eye region. More specifically, the N170 response was larger for fearful eye regions than for neutral and happy eye regions. Indeed, there is considerable evidence that fearful faces can amplify the response of the N170 across a variety of tasks and image manipulations, which suggests that the preferential processing of threats is relatively automatic and immune to available attentional resources [1,2,37,60]. Combining the emotion-irrelevant task and isolated eye region, the present N170 results replicate the findings observed in experiments that utilized full images of fearful faces [30,31]. With respect to the P3, a wealth of research has demonstrated that this component is involved in domain-unspecific emotional processing and is sensitive to task demands [3,44]. Some studies have shown a pronounced emotional effect of P3 in emotion discrimination tasks, gender decision tasks, and face detection tasks, with enlarged potentials for emotional vs. neutral faces [45,61]. Together, these findings indicate that the tasks entailed a deeper perceptual analysis involving varying degrees of P3 modulation. The heightened amplitude of the P3 component in response to happy vs. neutral and fearful eye regions may reflect the enhancement of perceptual encoding and sustained attention [44].

### 3.3. Experiment 3

#### 3.3.1. Behavioral Results

T2 accuracy was determined based on trials in which T2 was correctly reported and the confidence rating was labeled as “very sure” or “sure”. A two-way repeated measures ANOVA on T2 accuracy showed that the main effects of lag [*F*(1, 23) = 0.44, *p* = 0.51, *η_p_*^2^ = 0.02] and T1 valence [*F*(2, 46) = 1.52, *p* = 0.23, *η_p_*^2^ = 0.06], as well as their interaction [*F*(2, 46) = 0.49, *p* = 0.61, *η_p_*^2^ = 0.02] did not reach statistical significance.

#### 3.3.2. ERP Results

##### ERPs Related to the T1 Eye Region

**Figure 5:**
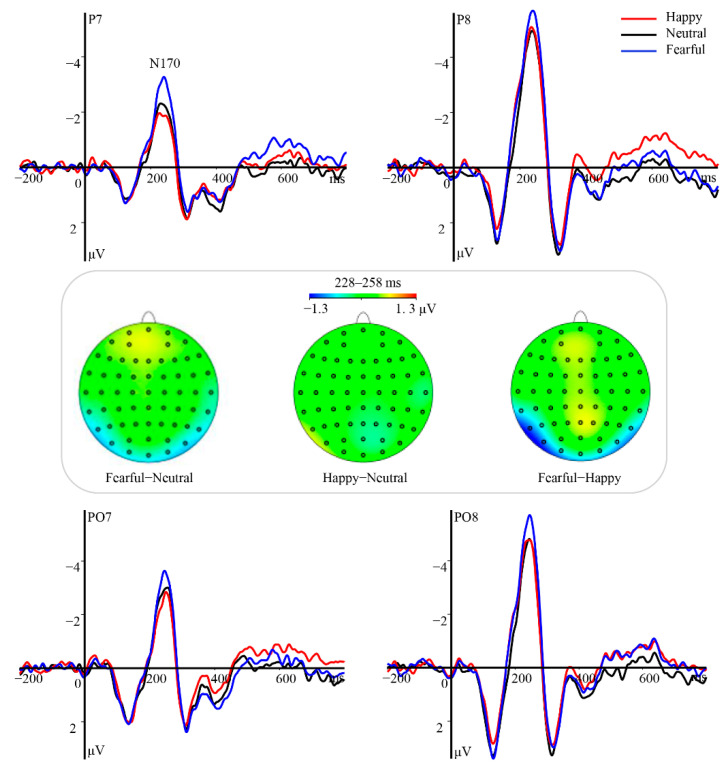
Main effect of emotional valence of eye regions on N170 amplitudes in Experiment 3. Top and bottom panels: Grand average ERP waveforms of N170 for happy (red line), neutral (black line), and fearful (blue line) eye regions at the indicated electrodes. Middle panel: Topographical difference maps depicting the effect of emotional valence in the eye region on the time window of N170.

Our findings indicated that the emotional valence of the eye region modulated the amplitude of the N170 component [*F*(2, 46) = 10.37, *p* < 0.001, *η_p_*^2^ = 0.31], which was higher for fearful stimuli (M ± SE, −4.26 ± 0.46 μV) than for happy (−3.39 ± 0.41 μV, *p* = 0.002) or neutral stimuli (−3.50 ± 0.45 μV, *p* = 0.006). However, the difference in amplitude between the latter two conditions was not significant (*p* = 1; Figure 5). We also observed a significant main effect of hemisphere [*F*(1, 23) = 59.99, *p* < 0.001, *η_p_*^2^ = 0.72]. Amplitudes were larger over the right hemisphere (−4.92 ± 0.51 μV) than over the left hemisphere (−2.51 ± 0.37 μV, *p* < 0.001), although we observed no significant interaction between emotional valence and hemisphere [*F*(2, 46) = 0.76, *p* = 0.47, *η_p_*^2^ = 0.03].

No significant effects of emotional valence [*F*(2, 46) = 1.00, *p* = 0.38, *η_p_*^2^ = 0.04] and hemisphere [*F*(2, 46) = 0.06, *p* = 0.94, *η_p_*^2^ = 0.01] were observed for the P3 component, and there were no significant interactions between the two [*F*(4, 92) = 0.65, *p* = 0.63, *η_p_*^2^ = 0.03].

##### ERPs Related to T2 Houses

In the analysis of N2 and P3 components in response to T2 houses, we observed no main effects of T1 valence [*Fs* < 1.24, *ps* > 0.29, *η_p_*^2^*s <* 0.05] or hemisphere [*Fs* < 3.83, *ps* > 0.07, *η_p_*^2^*s <* 0.14], and the interaction between the two did not reach statistical significance (*Fs* < 1.26, *ps* > 0.29, *η_p_*^2^*s <* 0.06).

To summarize, emotionally valent eye regions served as to-be-ignored distractors in Experiment 3. Neither the behavioral nor the ERP results indicated a significant AB effect, providing additional support to the notion of a task-dependent effect of emotional faces on AB [27,29]. Intriguingly, increases in N170 amplitude for fearful vs. neutral and happy eye regions were also observed. Previous studies have reported robust modulation of the N170 component by threat-related expressions in full face stimuli across a range of task and image manipulations [2,60,62]. Likewise, we observed increases in N170 amplitude when fearful stimuli were presented in Experiments 1 and 2 of the current study, regardless of task demands. The present results for N170 verify that threats are processed automatically and with priority in the early perceptual period.

## 4. Discussion

In the present study, we investigated the temporal dynamics underlying the processing of emotional valence in eye regions as well as the influence of different valences on AB using an ERP technique. In the expression discrimination task, happy eye regions were recognized more accurately than neutral eye regions. This finding is in accordance with consistent observations of advantageous processing for happy faces in previous studies [63]. Compelling evidence from cultural neuroscience also suggests that individuals in Eastern cultures fixate more on the eye region to identify facial expressions than those in Western cultures [12,64]. Therefore, it is reasonable to speculate that higher accuracy for happy stimuli reflected a genuine advantage in the recognition of happy faces. Moreover, houses presented as T2 stimuli following happy and fearful T1 stimuli were recognized more accurately than those presented after neutral T1 stimuli in the lag2 condition. Although T2 accuracy was higher than that traditionally reported (at chance level) [13], we believe that the AB effect actually appeared and was modulated by emotional T1 eye regions for two reasons. First, increases in T2 accuracy in the lag6 relative to the lag2 condition suggest limited availability of attentional resources for T2 house processing in the lag2 condition. Second, numerous studies have also reported accuracy levels higher than chance for emotional T2 presented within the AB period, supporting the notion that emotional stimuli receive prioritized access to limited cognitive resources [17,18].

### 4.1. The Neural Correlates of Eye Region Processing

The present results extend previous findings [6,39], as our analysis revealed functionally distinct stages involved in the processing of affective signals from the eye region. At the level of the N170, we observed consistent increases in amplitude for fearful stimuli when compared with responses for happy and neutral stimuli irrespective of task demands, confirming that the N170 is involved in the early affective encoding of the eye region and is especially sensitive to threatening information [19,40]. Several previous studies have reported that when compared with neutral faces, fearful faces can produce larger N170 amplitudes across a range of tasks and image manipulations, indicating a relatively automatic encoding of threat-related information [1,37,60]. Moreover, facilitated fearful face processing has been associated with higher emotional saliency in the eye region [39,40]. Thus, it is easy to understand why the N170 enhancement elicited by the fearful eye region was robust and task-independent. Additionally, the N170 amplitude elicited by happy stimuli was larger than that elicited by neutral stimuli only when attention was directed to the emotional properties of a target (Experiment 1). Such differentiation was not observed when attention was allocated to the non-emotional properties of a target (Experiments 2 and 3). This is consistent with the study of Wronka and Walentowska [65], who reported that noticeable N170 amplitude differences between happy and neutral faces were observed in an expression discrimination task but not in a gender discrimination task, which likely reflects voluntary mobilization of attention. Furthermore, recent empirical evidence suggests that both facial expressions and feature-based attention can modulate N170 amplitude [30,59,66]. Considering the decreased emotional saliency for happy stimuli in the emotionally irrelevant task as well as the effect of top-down attention, the differential modulation of N170 by stimuli depicting happy eye regions in the three tasks is reasonable. In this case, the emotional effect of N170 obtained may not only reflect relatively automatic processing of threats but also the modulation of top-down attention, adding to a recent wave of studies examining the susceptibility of N170 in the processing of emotionally laden facial expressions.

Regarding the emotional modulation of P3, stimuli depicting fearful and happy eye regions elicited larger amplitudes than those depicting neutral eye regions in the expression discrimination task (Experiment 1). When attention was explicitly directed toward the detection of an upright eye region (Experiment 2), we observed enhanced activity in response to happy compared to neutral eye regions. Furthermore, there was no significant main effect of emotional valence on the P3 component when these stimuli were consciously ignored (Experiment 3). These findings confirm that the emotional modulation of P3 is susceptible to task demands [31]. Indeed, there is increasing evidence supporting the idea that the P3 is involved in sustained and more elaborative processing of visual stimuli with high emotional relevance [19,59]. Thus, instruction to attend to a specific aspect of target faces may reliably impact the P3 response [44,58], and greater allocation of attentional resources to emotionally relevant facial features may lead to remarkable emotional modulations of P3. For instance, regardless of the level of trait anxiety, some studies have indicated that fearful faces increase the P3 response relative to that observed for neutral faces in expression discrimination tasks but not gender discrimination or line perception tasks [37,67,68]. Sun, Ren, and He [45] also reported increases in P3 amplitude for happy compared to neutral faces using a face detection task, and this effect has been interpreted as reflecting greater automaticity in happy face processing. Building upon these works, we speculate that differential emotional modulations of P3 in our three experiments may have been related to the synergy between emotional significance and top-down attention. Future studies should aim to uncover the extent to which emotional face processing at this post-perceptual stage is modulated by feature-based attention.

### 4.2. Effect of T1 Emotional Task Relevance on AB

The present study employed three attention tasks to gradually decrease the attention to emotion-related information in T1 (emotion discrimination, eye detection, eyes ignored). Our results indicated that happy and fearful T1 stimuli attenuated the AB only in the emotion discrimination task. That is, when participants were asked to discriminate the expression of the T1 eye regions, accurate identification of happy and fearful eye regions resulted in significantly higher accuracies for subsequent T2 houses in the lag2 condition when compared with rates observed in the neutral condition. However, the valence of the eye region did not significantly modulate accuracies for T1 or T2 in either the eye-detection task (Experiment 2) or eyes-ignored task (Experiment 3). These observations support the notion that the emotional modulation of AB by face stimuli is task-specific [29,69]. Previous studies have reported that threat-related faces presented as T1 remarkably hamper the recognition of T2 and result in an enhanced AB, whereas happy T1 faces facilitate T2 recognition and attenuate the AB [23,24,29]. These findings are partially in contradiction with our results. The differences in affective intensity and potential encoding mechanisms may explain this discrepancy. Although no differences in the magnitude of the threat superiority effect are observed between fearful stimuli depicting the full-face and those depicting the eye region [9], their abilities to capture and hold attention are not the same. Indeed, the emotional intensity expressed by the eye region may be lower than that expressed by the full face [70], and it is possible that attentional disengagement from the fearful eye region is easier, resulting in more elaborated processing of subsequent T2 and an attenuated AB. The Lateral Inhibition, Face Template, and Eye Detector model indicates that both face-sensitive and eye-sensitive neurons are involved in the processing of the eye region, although activation of eye-sensitive neurons is inhibited during full-face processing [34,35,36].

Of significance, our ERP data provide the first piece of evidence regarding the neural correlates of the influence of T1 emotional task relevance on AB, as reflected by the N2 and P3 components for T2 houses at lag2 in Experiment 1. More precisely, when T2 houses were preceded by happy or fearful T1 stimuli, N2 and P3 amplitudes over the right hemisphere were larger than those observed for stimuli preceding by a neutral T1. A series of ERP studies on AB has demonstrated that the N2 and P3 are involved in early selective attention and representation consolidation of the T2 stimuli in working memory, respectively [48,50,71]. Kennedy, Rawding, Most, and Hoffman [47] reported attenuation of the target-related N2 and P3 when preceded by a negative distractor (task-irrelevant), relative to the neural distractor condition. Thus, the negative distractor may suppress attentional capture and sustained elaborate processing. In parallel with the behavioral results, we speculate that the enhancement of N2 and P3 for T2 houses reflects the greater allocation of attentional resources to T2 processing due to facilitated processing of happy and fearful T1 eye regions.

Given the obvious perceptual differences in eye regions with emotionally valent information, one may argue that the effects of emotion and AB discussed above are driven by physical features of the eye region rather than its affective content. Nevertheless, no significant emotional effect of P1 was observed in any of our three experiments. In fact, as the P1 component is assumed to be sensitive to the low-level properties of a stimulus [62], the lack of an emotional P1 effect (Table A1 in Appendix) may allow us to exclude the influence of low-level eye features on the results to some degree. Furthermore, one of our previous studies adopting the same eye region images for T2 found that both happy and fearful eye region stimuli can reduce the AB [17], indicating that the visual saliency was similar for the two stimulus types. If the N170 response was driven by visual salience of the eye regions, comparable amplitudes would have been observed for fearful and happy trials, in contrast to the pattern observed in our study (fearful > happy). Other studies have also demonstrated no effect of the perceptual saliency of the visual stimuli on AB [23,72]. Although these results suggest that the pronounced emotional effects observed in our study are not related to perceptual saliency, future studies should address this issue via strict manipulation of perceptual properties in the eye region.

The present study had several limitations. First, to avoid the effects of practice and fatigue, a mixed experimental design was used. The task demand of the T1 was manipulated between experiments, and the valence of eye regions and T1–T2 lag were used as within-subject factors. Nevertheless, the influence of individual differences in selective attention [73,74] and the imbalance in the number of male and female participants across the three experiments may restrict the generalizability of our findings regarding the effects of task demands on AB. Second, given the influence of individual differences in information transmission in the eye region, there were only two pictures for each type of T1 stimuli. This manipulation may have increased the familiarity of each stimulus picture, leading to reduced task difficulty and the presence of a ceiling effect. Some related studies have found that processing familiar faces requires less attention. Thus, presenting familiar images as T2 may have attenuated the AB effect relative to that for unfamiliar faces [75,76]. Future studies with a larger quantity of stimuli materials and a within-subject design are necessary to fully illustrate the temporal dynamics of the task-relevant effects of emotional T1 on AB.

## 5. Conclusions

The current findings indicate that feature-based attention to T1 eye regions differentially modulates the emotional processing of eye regions and the identification of subsequent task-relevant targets. The face-sensitive N170 component is particularly susceptible to fearful signals derived from the eye region and is immune to task demands, reflecting the relatively automatic processing of threatening information at an early sensory stage, while emotional effects on P3 largely depend on the task demands. Furthermore, our results suggest that the preferential processing of emotional eye regions can enhance attentional allocation to subsequent T2 and elicit larger N2 and P3 responses when an attentional task is explicitly related to T1 emotional processing, leading to reduced AB. Together, these findings demonstrate that attentional resources are required for the processing of emotion-laden eye regions, and their emotional modulations on temporal attention are sensitive to the task set.

## Data Availability

The data are available from the corresponding author upon reasonable request.

## Appendix A

In order to see the influence of physical features of emotionally valenced eye region on the P1 component, three additional two-way repeated measures ANOVAs with valence and hemisphere as within-subject factors were conducted. The selection criteria of time window and electrode are similar to the components mentioned above. For the P1, six electrodes of O1, O2, PO7, PO8, P7, and P8 with the time window of 113–143 ms were analyzed. Neither the main effects of hemisphere and valence nor their interaction reached significance.

**Table A1:** P1 amplitudes for happy, neutral, and fearful eye regions under different tasks. Means are presented with standard error in parentheses.

	Happy	Neutral	Fearful
Emotion discrimination task	2.07 (0.31)	2.01 (0.31)	1.86 (0.28)
Eyes detection task	1.78 (0.24)	1.88 (0.21)	1.84 (0.26)
Eyes to be ignored task	1.82 (0.34)	2.07 (0.28)	2.04 (0.29)
